# H5N1: A Cautionary Tale

**DOI:** 10.3389/fpubh.2014.00117

**Published:** 2014-08-12

**Authors:** Susan A. Ehrlich

**Affiliations:** ^1^Judge (retired), Arizona Court of Appeals, Phoenix, AZ, USA

**Keywords:** H5N1, dual use research of concern, biosecurity, biosecurity policy, bioterrorism, National Science Advisory Board for Biosecurity

The history of the research with the highly pathogenic avian influenza virus H5N1 and the publication of the results of that research is reminiscent of the Buddhist “Parable of the Blind Men and the Elephant.” In the parable, the Buddha relates a story of a raja who, when confronted with disputatious scholars, gathered blind men and presented each man with a part of an elephant, telling him “here is an elephant.” The raja then asked each man to describe the animal. As each man explained “what sort of thing is an elephant,” the men began arguing about whether the likeness was to a pot or a basket or a pillar, etcetera, eventually coming to blows over the question and prompting the Buddha to observe: “for, quarreling, each to his view they cling. Such folk see only one side of a thing”^1^. Fortunately, the H5N1 debates have not led to blows but instead have yielded important provocative discussions about the importance of the research and the issues and implications of communicating the results of the research (1). Unfortunately, there still is no consensus regarding the manner of the oversight of life-sciences dual-use research of concern (DURC).

In the fall of 2011, the National Science Advisory Board for Biosecurity (NSABB), a United States Government Advisory Committee, was charged with reviewing two manuscripts describing research funded by the National Institute of Allergy and Infectious Diseases (NIAID) in which genetically modified H5N1 was shown to have the potential for respiratory transmission between ferrets. The primary author of the manuscript intended for publication in *Nature* was Yoshihiro Kawaoka, Ph.D., DVM, of the University of Wisconsin-Madison and the University of Tokyo; the primary author of the manuscript intended for publication in *Science* was Ron A.M. Fouchier, Ph.D. of the Erasmus Medical Center in Rotterdam. The mandate was the result of the manuscripts having been shown to the White House National Security Staff, which referred its concerns that the publication of the manuscripts might have biosecurity implications to the Department of Health and Human Services, which in turn asked the NSABB for its recommendations.

The NSABB formed an H5N1 Working Group. The Working Group and the Board spent hundreds of hours discussing the issues presented by and swirling around the manuscripts, all of which conversations and meetings were closed due to the very nature of the purpose for which the NSABB had been convened, i.e., biosecurity concerns posed by the publication of the manuscripts as well as the interests of the authors and publishers that the manuscripts not be made public prematurely. On December 20, 2011, the NSABB announced its recommendation that the authors’ “general conclusions highlighting the novel outcome be published but that the manuscripts not include the methodological and other details that could enable replication of the experiments by those who would seek to do harm” (2).

Subsequently, the authors’ revised manuscripts were submitted to the NSABB and discussed at a meeting on March 29 and 30, 2012. This assembly included the perspectives of the governments of the Kingdom of the Netherlands and Japan^2^ and from meetings sponsored by the World Health Organization and the American Society for Microbiology. Additionally, the “United States Government Policy for Oversight of Life Sciences Dual Use Research of Concern” was issued at the end of the first day of the 1^1/2^-day conference (3). After a robust debate among its voting and ex officio members, which ex officio membership included NIAID’s director, the NSABB unanimously recommended that the revised Kawaoka manuscript should be “communicated in full” (4). It also recommended, but in a 12-to-6 decision, “the communication of the data, methods, and conclusions presented in [the] revised Fouchier manuscript”^3^.

The NSABB path of H5N1 review was to varying degrees rocky from beginning to end. Some of the confusion could have been avoided; some of the commotion could not have been escaped. Pertinent questions include why the process was disordered, how confusion could have been or be avoided and whether another mechanism should replace that of the NSABB.

The NIAID-funded studies undoubtedly should have been highlighted for biosecurity concerns well before the results of the H5N1 research were being readied for publication. Questions whether the research might have the potential to be DURC should have been considered during the design, execution, and reviews of the research, and there should have been in place a communication plan given the potential for novel results. From early on, the research clearly was within the seven categories of experiments that could constitute DURC and warrant particular scrutiny according to the 2004 National Research Council report *Biotechnology Research in an Age of Terrorism* (5) and the 2007 NSABB report *Proposed Framework for the Oversight of Dual Use Life Sciences Research* (6). As a consequence of this failure, much of the NSABB discussion centered around the potential for the research to be used for malevolent purposes – the nature and results of the research, its complexity in terms of how readily the research could be replicated, by whom it could be reproduced and made more dangerous, what facilities and what conditions would be necessary, and how it could be disseminated – and a crucial question of whether the benefits for public health outweighed the risks.

As noted, the manuscripts necessarily were kept confidential because of the biosecurity concerns and the interests of the authors and publishers, but before the review of the revised manuscripts, the Dutch government invoked European export-control legislation as well. The secrecy surrounding this review process led to various ill-founded assumptions, misunderstandings, commentaries by individuals who did not know how the matter came to the NSABB and/or would not have read the manuscripts, leaks – some of which were not accurate reflections of the discussions, and reporting errors, all of which harmed the NSABB’s credibility despite the NSABB lacking the ability to respond without violating the confidentiality to which its members were sworn. Some kind of equilibrium between transparency and consensus versus security thus was an important issue for the NSABB when discussing how and to whom the results of the H5N1 research could be communicated, but a satisfactory balance, while ardently sought by the NSABB, was not found.

Ultimately, the NSABB proved not to be an exemplary model and instead showed that a new, autonomous advisory commission must be created as its substitute. The better practical model would have two components: requirements for the federal funding agency and for a federally funded researcher and institution, and a Presidential Commission for the Oversight of Dual Use Life Sciences Research established by an Executive Order.

The first element would be fivefold: (a) a requirement that the federal departments and agencies have personnel with sufficient expertise to screen research proposals for DURC potential with the concomitant requirement that if such potential exists, the researcher be asked to consider modifying the research to reduce risk; (b) a means to make sure that the facilities are adequate that the researcher and laboratory staff are knowledgeable about DURC issues and engage in a continuing review to minimize any necessary risk, and that the institution has a communication plan regarding novel techniques and/or novel results; (c) the mandatory education of each researcher in biosecurity in conjunction with biosafety for the purpose of recognizing and addressing DURC issues; (d) the requirement that the researcher attest to a review of the research for DURC potential at the beginning and on each occasion of a funder’s review; and (e) the requirement that each institution have a committee that includes the institution’s responsible official, additional experts and community members to review the conduct of research identified as having DURC potential.

The second element would be the creation of a Presidential Commission to serve as a truly independent expert advisory group. Its voting membership would be appointed and include individuals nominated by the federal departments and agencies that conduct, support, or have interests in life-sciences research, including the intelligence community, so as to be comprised of persons reflecting a diversity of scientific and other relevant expertise and interests, including that of the public, in order to provide divergent perspectives. The Commission would have a staff and budget separate from any federal department or agency, and it would be able to convene itself and set its own agenda. Its members would have access to security information, and it would be able to call upon experts from outside government. Ex officio members would represent the interested federal departments and agencies with the caveat that the department or agency that funded the research would be limited in participation in any discussion of that research because of the inherent conflict of interest possessed by a department’s or agency’s stake in promoting the communication of research that it has found sufficiently important to fund.

The Commission would be the authoritative voice to the United States Government. Federal departments and agencies would be expected to refer to its expertise in decisions whether to fund research identified as potential DURC at the outset and during continuing reviews; by this means, the Commission could provide needed consistency and integrated approaches among the departments and agencies. The Commission also would be available to an institution, whose DURC-review committee refers queries to it, and it also would be available to the editors and publishers of scientific journals who now by default are the arbiters of whether and what data and analyses are published. As would be true of the institutional committee, the Commission’s responsibilities would not be unduly burdensome because as a practical matter, very little scientific research constitutes DURC.

Additionally, the Commission should undertake other duties: provide educational materials to institutions and vigorously promote their use; propose federal standards for personnel reliability; recommend approaches regarding the communication of the results of DURC research along the continuum of full disclosure to government classification, including restricted access; and undertake a review of the plethora of the federal statutes, regulations, rules, guidelines, and policies for the purpose of organizing the existing regulatory cacophony, which jumble burdens and discourages scientific research.

As a general, undisputed principle, the unrestricted dissemination of the results of scientific research is critical for the progress of science. When this must be compromised for reasons of national security, there has to be a means by which the results can be shared with trusted and responsible researchers and institutions. Among the unresolved issues, however, is who is responsible for the decision? Should the Commission’s guidance control the agency’s or department’s funding decision subject to an appeal to the Cabinet-level officer or the National Security Council? By what mechanism will compliance be enforced? The circumstances of the publication of the results of the H5N1 research will be repeated in a multitude of circumstances, e.g., ongoing gain-of-function research with highly pathogenic avian influenza viruses, a new botulinum toxin serotype. Only from an increasing mindfulness of biosecurity issues and DURC, in particular, will come a legitimate approach – and a legitimate approach must be found – to safeguarding public health, safety, and security without compromising the essential vitality of the scientific enterprise.

## Conflict of Interest Statement

The author declares that the research was conducted in the absence of any commercial or financial relationships that could be construed as a potential conflict of interest.

## Footnotes

^1^*The Udana* 68–69

^2^Other countries expressed their apprehensions about a precedent that the H5N1 research results would not be shared with them although it was they who had shared samples of those viruses with the researchers.

^3^The author was one of the six members who did not concur with the majority’s recommendation with reference to the Fouchier manuscript.
